# Widespread deployment of the human CD38 ADP-ribosyl cyclase fold in antibacterial and anti-eukaryotic polymorphic toxins

**DOI:** 10.1016/j.jbc.2025.110775

**Published:** 2025-09-27

**Authors:** Julius Martinkus, Laurent Terradot, Dukas Jurėnas, Eric Cascales

**Affiliations:** 1 Laboratoire d'Ingénierie des Systèmes Macromoléculaires (LISM), Aix-Marseille Université, CNRS, UMR 7255, Marseille, France; 2 Laboratory of Molecular Microbiology and Structural Biochemistry, Institut de Biologie et Chimie des Protéines (IBCP), Université Claude Bernard, CNRS, UMR 5086, Lyon, France; 3 Bacterial Genetics and Physiology, Faculté des Sciences, Université Libre de Bruxelles (ULB), Gosselies, Belgium; 4 WEL Research Institute, Wavre, Belgium

**Keywords:** type VI secretion, polymorphic toxin, NAD^+^, ADP-ribosyl cyclase, CD38

## Abstract

Bacterial polymorphic toxins are modular weapons that mediate intermicrobial competition and host interactions by delivering diverse cytotoxic domains through specialized secretion systems. Here, we identify and characterize a novel toxin domain in *Pantoea ananatis* that displays remarkable structural and functional conservation with the human enzyme CD38. This bacterial toxin, fused to a type VI secretion system (T6SS) PAAR domain, harbors a C-terminal ADP-ribosyl cyclase (ARC) domain that hydrolyzes NAD^+^ and NADP^+^*in vitro* and *in vivo*, leading to growth inhibition in both bacterial and eukaryotic cells. The 1.6-Å resolution structure of ARC reveals that it adopts a globular fold nearly identical to the human CD38 ADP-ribosyl cyclase, with key catalytic residues conserved. Comparative genomics reveals that CD38-like ARC domains are widespread in bacteria, fused to diverse delivery modules including T6SS, T7SS, and CDI systems. Functional assays demonstrate that these domains act as NAD-depleting toxins, with cross-immunity observed between non-cognate toxin–immunity pairs. Taken together, our findings identify a bacterial NAD^+^ hydrolase fold with strong similarity to human CD38 and define a novel class of metabolic toxins, expanding the functional scope of polymorphic effectors and illustrating how enzymes can be co-opted for microbial warfare.

Bacteria live in densely populated, competitive environments where access to nutrients and space is limited (1, 2, 3, 4, 5). To thrive, many species have evolved potent weapons to inhibit or eliminate their rivals (6, 7, 8, 9, 10, 11, 12, 13, 14). Among the most versatile of these weapons are polymorphic toxins (PT), which are modular, multidomain proteins that are delivered into neighboring cells to sabotage essential processes and cellular functions (10, 15, 16).

These toxins are often secreted through sophisticated nanomachines, such as the type IV (T4SS), type V (T5SS), type VI (T6SS) or type VII (T7SS) secretion systems (6, 8, 12, 17, 18, 19, 20, 21, 22, 23), and are typically accompanied by immunity proteins that protect the producing bacterium from self-intoxication (24, 25). The diversity and adaptability of polymorphic toxins have made them central players in interbacterial warfare, microbial community structuring, and even host interactions (15).

A defining feature of polymorphic toxins is their modularity. These proteins share a conserved N-terminal trafficking domain fused to highly variable C-terminal “effector” domains, which confer toxic activity (10, 15, 16). Over evolutionary time, this modularity has enabled the shuffling and specialization of toxin domains, resulting in a vast repertoire of enzymatic activities, including nucleases, peptidases, lipases, or NAD-targeting enzymes (16). Increasingly, it has become clear that bacteria do not solely evolve new toxic domains *de novo*; they also select, co-opt, and repurpose host-like proteins for bacterial warfare (15, 26, 27).

One of the most intriguing examples is toxin mimicry, *i.e.*, the occurrence of eukaryotic-like domains in bacterial toxins, which can closely resemble host enzymes in structure and function (26, 28, 29). Such similarities may arise from shared biochemical constraints, convergent evolution or ancient common ancestry. This results in bacterial effectors that perform activities analogous to those of host proteins and are used in competition, colonization, or immune evasion (28, 29). Notably, several bacterial toxins appear to mimic or structurally resemble eukaryotic proteins involved in key cellular processes, such as actin polymerization, cell signaling, and nucleotide metabolism (26, 28, 30, 31).

In this study, we describe the discovery of a novel toxin domain in *Pantoea ananatis* that exemplifies this strategy of molecular hijacking. The toxin, encoded within a T6SS *vgrG*/*hcp*/*paar* island, harbors an N-terminal Proline-Alanine-Alanine-Arginine (PAAR) trafficking domain, putative transmembrane helices and a C-terminal domain with strong structural and functional similarity to the human enzyme CD38 (Cluster of Differentiation 38), a multifunctional ADP-ribosyl cyclase (ARC) involved in immune cell activation and regulation of calcium intracellular levels and signaling (32, 33, 34, 35, 36). We show that this bacterial toxin, designated ARC^tox^, shares the catalytic residues, enzymatic activity, and overall fold of CD38, yet functions as a cytotoxic effector capable of depleting NAD^+^ and NADP^+^ in both bacteria and eukaryotic target cells, suggesting a dual role in microbial competition and host interaction. By tracing the distribution of ARC-like domains across bacterial genomes, we reveal that CD38-like folds are found in a wide range of bacteria and fused to diverse secretion and delivery systems, highlighting the remarkable evolutionary plasticity of PT.

Together, our findings indicate that CD38-like NAD^+^ hydrolase domains are not restricted to eukaryotes but also occur as components of bacterial polymorphic toxins, where they can be used in bacterial warfare. This expands the known functional repertoire of PT and provides insight into how similar enzymatic folds can be employed for distinct biological roles.

## Results

### The C-terminal domain of the *P. ananatis* PAAR PANA_2924 protein is toxic when expressed in the *Escherichia coli* or yeast cytosol

The plant pathogen *P. ananatis* strains LMG 2665^T^ and DZ-12 use a type VI secretion system (T6SS) to promote virulence in onion plants and to outcompete various bacterial species, including *E. coli* (37, 38). Other *Pantoea* species encode up to three distinct T6SS gene clusters, each containing all essential structural components, as well as a variety of *vgrG*, *hcp*, and *PAAR* islands (39, 40, 41). In addition to T6SS needle components, these genomic islands often encode effector proteins, including the recently characterized TseG nuclease (38).

**Figure 1:**
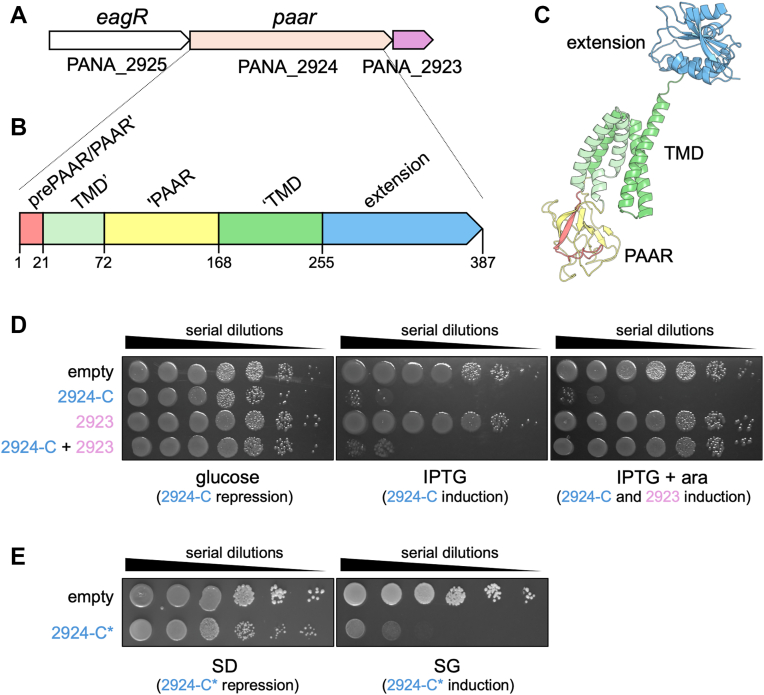
***Pantoea ananatis* PANA_2924 C-terminal domain is toxic in *E. coli* and yeast cells.***A*, schematic representation of the *P. ananatis PANA_2925-PANA_2923* PAAR island. Genes encoding the EagR chaperone (PANA_2925), the specialized PAAR protein (PANA_2924), and a protein of unknown function (PANA_2923) are depicted in *white*, light flesh and *pink*, respectively. *B and C*, schematic representation (*B*) and AlphaFold3 structural model (*C*) of the PANA_2924 specialized PAAR protein, highlighting the different domains and their boundaries: PAAR domain (PAAR’ or prePAAR in *light red*, and ‘PAAR in *yellow*), trans-membrane domain (TMD’ and ‘TMD in *light green* and *green*, respectively), and C-terminal extension (*blue*). *D*, toxicity assay in the heterologous host *E. coli.* Overnight cultures of *E. coli* cells expressing the PANA_2924 C-terminal extension (2924-C) from the low-copy vector pNDM220, PANA_2923 (2923) from the pBAD33 vector, or both (2924-C+2923) were serially diluted (10^-^^0^ to 10^-6^) and spotted on LB agar plates, supplemented with 1% of glucose (*left* panel, 2924-C repression), with 0.05 mM of IPTG (*middle* panel, 2924-C induction), or with 0.05 mM of IPTG and 1% of L-arabinose (*right* panel, 2924-C and 2923 induction). *E*, toxicity assay in yeast. Overnight cultures of W303 cells expressing the attenuated PANA_2924-C∗ mutant from the pRS416_Gal1 vector were serially diluted and spotted on SD (*left* panel, 2924-C repression) or SG (*right* panel, 2924-C induction) medium.

In the *P. ananatis* reference strain LMG 20103, one such *PAAR* island comprises three genes encoding a putative EagR-family chaperone (PANA_2925, GeneBank Identifier (GI): ADD78092.1), which usually binds to and protects effector transmembrane domains (42, 43, 44, 45), a polymorphic PAAR protein with an extended C-terminal region of unknown function (PANA_2924, GI: ADD78091.1), and a small protein (PANA_2923, GI: ADD78090.1) (Fig. 1*A*). This gene organization resembles canonical chaperone-dependent polymorphic toxin operons, where a central toxin gene fused to a trafficking domain is flanked by a chaperone and an immunity protein.

Structural predictions using AlphaFold3 and BLASTp searches revealed that PANA_2924 is comprised of three distinct domains: a split PAAR domain (residues 1–21 (PAAR’ or pre-PAAR) and 72 to 168 (‘PAAR)), which could associate with the T6SS needle spike, a five-helix bundle (residues 22–71 and 169–249), and a globular C-terminal extension (PANA_2924-C, residues 255–387) (Fig. 1, *B* and *C*). It is noteworthy that the PAAR domain is interrupted by two helices from the central bundle (Fig. 1*B*), yielding PAAR’ (or prePAAR) and ‘PAAR regions, as previously observed (42, 44).

To test whether the C-terminal extension corresponds to a toxin module, PANA_2924-C was cloned under the control of the P_*lac*_ promoter into the low-copy pNDM220 vector, and its toxicity was tested in *E. coli*. While PANA_2924-C was not toxic when its expression was repressed by the presence of glucose, induction with IPTG led to strong growth inhibition, indicating antibacterial activity in the *E. coli* cytosol (Fig. 1*D*). As the *P. ananatis* T6SS is involved in virulence toward onions (37, 38), we tested whether the PANA_2924-C was also toxic to eukaryotic cells. The same domain was expressed in *Saccharomyces cerevisiae* from the single-copy pRS416 plasmid. We were, however, unable to clone the PANA_2924-C into pRS416, likely due to its toxicity and plasmid leakiness in *E. coli* during the cloning procedure, but we obtained a clone (PANA_2924-C^∗^, see Experimental Procedures) that is likely attenuated in its toxicity. Yeast growth was significantly inhibited upon induction of PANA_2924-C^∗^ expression (Fig. 1*E*), demonstrating that the C-terminal extension of the PANA_2924 specialized PAAR protein has dual antibacterial and anti-eukaryotic activity.

### PANA_2923 encodes an immunity protein that neutralizes PANA_2924 toxicity through protein-protein interaction

To determine whether the downstream gene, PANA_2923, encodes a cognate immunity protein conferring protection against PANA_2924, it was cloned under the *ParaBAD* promoter in the pBAD33 vector and co-expressed with the toxic PANA_2924 C-terminal domain. Co-expression fully rescued *E. coli* growth (Fig. 1*D*), demonstrating that PANA_2923 neutralizes the toxin’s activity.

**Figure 2:**
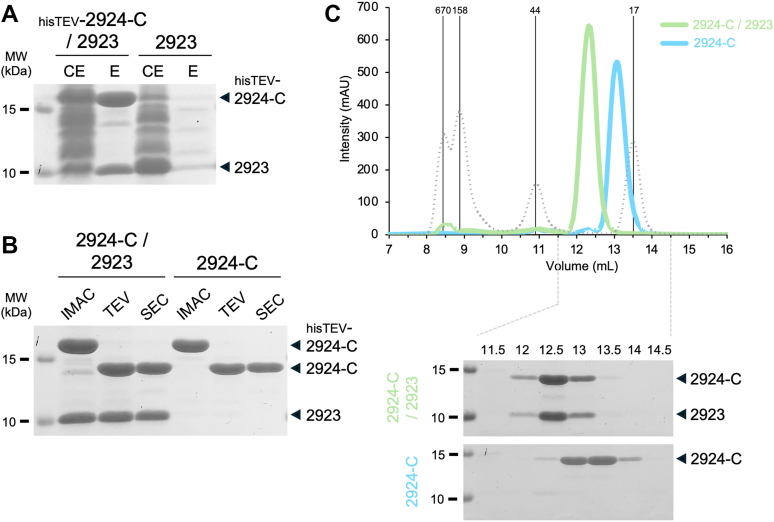
**PANA_2924 C-terminal extension forms a complex with PANA_2923.***A*, Pull-down assay. Soluble extracts of *E. coli* BL21(DE3) cells producing 6× His-TEV-2924-C and untagged PANA_2923, or untagged PANA_2923, were subjected to pull-down by immobilized metal affinity chromatography (IMAC). The cell extract (CE) and eluted material (E) were separated by 15%-acrylamide SDS-PAGE and stained with Coomassie *blue*. The positions of the proteins are indicated on the *right*. Molecular weight markers (in kDa) are indicated on the *left*. *B*, purification of 2924-C/2923 complex and 2924-C alone. Soluble extracts of *E. coli* BL21(DE3) cells producing 6× His-TEV-2924-C and untagged PANA_2923 were subjected to IMAC purification, and gel filtration (SEC) after cleavage by the TEV protease. For 2924-C alone, the complex immobilized by IMAC was washed with 8M urea to dissociate and discard untagged 2923, prior to elution, TEV cleavage, and SEC. The fractions after IMAC, TEV cleavage and SEC were separated by 15%-acrylamide SDS-PAGE and stained with Coomassie blue. The positions of the proteins are indicated on the *right*. Molecular weight markers (in kDa) are indicated on the *left*. *C*, gel filtration analysis. The PANA_2924-C/PANA_2923 complex (*green line*) and the PANA_2924-C toxin alone (*blue line*) obtained after affinity purification and TEV cleavage were subjected to size-exclusion chromatography on a Superdex 75 Increase 10/300 Gl column. The elution profile of molecular weight standards is shown as dotted line and the molecular weight of each of the standard is indicated on *top* (in kDa). Below the graph are shown the 15%-acrylamide SDS-PAGE and Coomassie blue staining analyses of selected SEC fractions encompassing the 2924-C/2023 (*green, upper* panel) and 2924-C (*blue, lower* panel) peaks. The calibration curve, linear regression, R^2^ value and experimental molecular weights of the complex and toxin alone are shown in Figure S1.

Co-purification assays using affinity chromatography confirmed a direct interaction between untagged PANA_2923 and 6 × His-TEV-tagged PANA_2924 C-terminal domain (Fig. 2*A*). This complex was stable enough to resist TEV cleavage and size-exclusion chromatography (SEC) (Fig. 2*B*). However, the complex could be separated upon addition of 8M urea in the affinity chromatography wash buffer, which allowed the purification of the PANA_2924-C domain alone (Fig. 2*B*). The isolated domain and the complex were eluted from the Superdex 75 SEC column with a volume of 13.1 ml and 12.3 ml (Fig. 2*C*). Based on the calibration curve (Fig. S1), these elution volumes are consistent with molecular weights of 19.2 and 27.3 kDa for PANA_2924-C alone (theoretical mass: 16.5 kDa) and PANA_2924-C/PANA_2923 (theoretical mass: 25 kDa), respectively, suggesting a 1:1 stoichiometry between the toxin and its immunity protein.

### PANA_2924 C-terminal domain structure reveals an ADP-ribosyl cyclase fold

**Figure 3:**
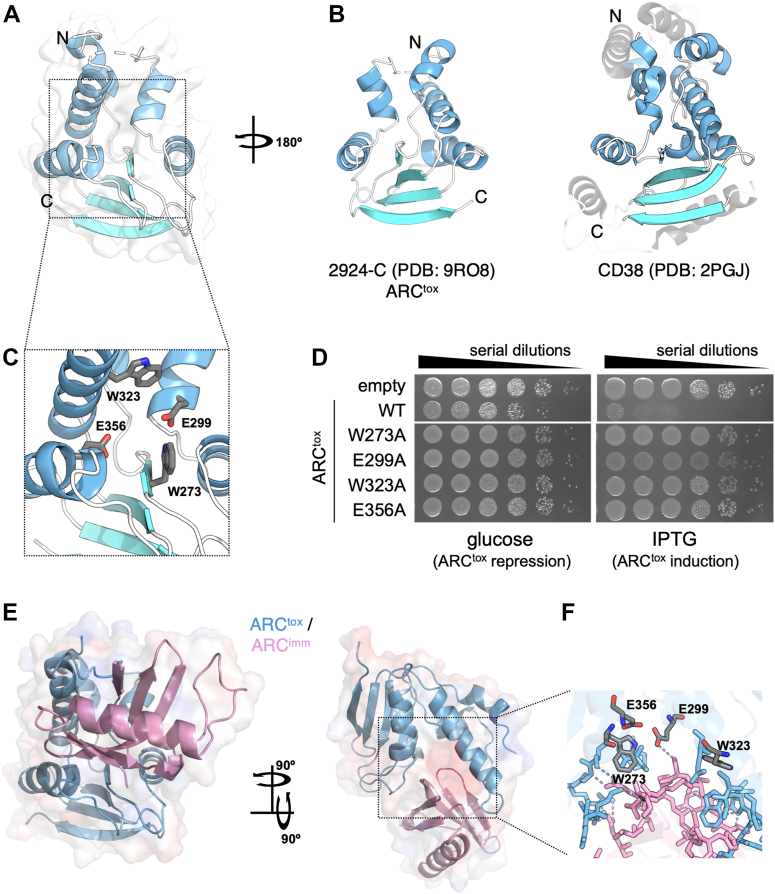
**PANA_2924 C-terminal extension displays an ADP**-**ribosyl cyclase (ARC) fold comparable to human CD38, with a typical catalytic cleft blocked by the PANA_2923 immunity protein.***A*, ribbon representation of the 1.6-Å resolution crystal structure of PANA_2924 C-terminal extension. α-helices are depicted in *light blue* whereas β-strands are shown in *cyan*. The surface structure is shown in *grey* transparent. N, N-terminus; C, C-terminus. *B*, structural comparison between *P. ananatis* PANA_2924 C-terminal extension (this work) and human CD38 protein (PDB: 2PGJ) in the same orientation. Conserved secondary structures are colored as in panel *A*. N, N-terminus; C, C-terminus. The PANA_2924-C structure is shown rotated 180° relative to the vertical plane compared with panel A. The amino-acid alignment of PANA_2924-C and human CD38 is shown in Figure S3*A*. *C*, close-up view of the PANA_2924-C putative active site, showing potential key residues as *grey* sticks. A comparison of PANA_2924-C and CD38 NAD^+^-binding pockets, including catalytic residues, is shown in Figure S3*B*. *D*, toxicity assay in the heterologous host *E. coli.* Overnight cultures of *E. coli* cells expressing the wild-type (WT) or mutated ARC^tox^ from the low-copy vector pNDM220 were serially diluted (10^-1^ to 10^-6^) and spotted on LB agar plates, supplemented with 1% of glucose (left panel, ARC^tox^ repression), or 0.05 mM of IPTG (*right panel*, ARC^tox^ induction). *E*, ribbon representations of the AlphaFold3 prediction model of the ARC^tox^/ARC^imm^ complex (ipTM score = 0.92). The toxin domain is shown in *blue*, whereas the immunity is shown in *pink*, within the complex’s surface representation colored based on residue electrostatic properties (from *blue* (positive) to *red* (negative)). pLDDT and PAE confidence scores of the ARC^tox^/ARC^imm^ complex are presented in Figure S4. *F*, contact site between ARC^tox^ and ARC^imm^ highlighting the ARC^imm^ loop penetrating into the ARC^tox^ catalytic cleft. The side chains of the ARC^tox^ catalytic residues are shown in *grey*. Residues within 4 Å of the ARC^tox^ and ARC^imm^ interface are shown as *sticks*, and *dotted grey lines* indicate potential polar contacts.

The purified complex of the PANA_2924 C-terminal domain and its PANA_2923 immunity was subjected to crystallization trials without success. Crystallization of the PANA_2924 extension was, however, achieved after dissociating the immunity protein from the complex by adding urea to the affinity chromatography washing buffer, followed by refolding and SEC purification of the isolated toxin (Fig. 2*B*). An X-ray diffraction dataset was collected, and the structure was solved by molecular replacement using the AlphaFold3 model, followed by refinement to a resolution of 1.6 Å (Fig. 3*A*, Table S1). PANA_2924 crystallized in the space group P2_1_2_1_2_1_, with two molecules per asymmetric unit. These two molecules adopt similar conformations (Fig. S2). However, while the first chain could be modeled from residues 252 to 385, missing a flexible loop from 311 to 317, the second chain could be modeled from residues 250 to 385, missing a different flexible loop (amino acids 345–349) (Fig. S2). The structure reveals a globular fold with two lobes, consisting of a four-stranded β-sheet covered by a helical cap composed of five α-helices (Fig. 3*A*). A search for structural homologues using DALI (46) showed that the PANA_2924 C-terminal extension displays a fold similar to the human protein CD38 (Z-score: 8.6). Although the overall identity of the PANA_2924-C and CD38 domains retrieved using DALI is relatively low (26.3%) (Fig. S3*A*), the two domains are indeed structurally similar (Fig. 3*B*). CD38 is a multifunctional protein: CD38 is a receptor at the surface of immune cells, responsible for the activation of T cells to produce cytokines (47). CD38 has also ADP-ribosyl cyclase (ARC) activity: It hydrolyzes NAD^+^ to generate cyclic ADPribose (cADPr), a messenger mediating various physiological functions in eukaryotic (notably involved in calcium signaling) and bacterial cells (33, 36, 48, 49, 50). Importantly, key catalytic residues of the CD38 ARC domain—two glutamates and two tryptophans located in the cavity between the β-sheet and the α-helical cap (36, 51, 52); Fig S3*B*)—are conserved in the PANA_2924 ARC toxin (hereafter named ARC^tox^) (Fig. 3*C*). These residues (W273, E299, W323, E356) form a putative active site. Indeed, substitution of any of the ARC^tox^ putative catalytic residues, W273, E299, W323, and E356, by alanines abolished ARC^tox^ activity in *E. coli* (Fig. 3*D*). In agreement with this information, the AlphaFold3 model of the ARC^tox^ with its immunity protein PANA_2923 (ARC^imm^), predicted with high confidence (Fig. S4), suggests that ARC^imm^ interacts extensively with the ARC^tox^ catalytic cleft (interface area of 1130 Å), with a loop projecting into the active site (Fig. 3, *E* and *F*).

### ARC^tox^ hydrolyzes NAD^+^ and NADP^+^*in vitro* and *in vivo* but does not produce cADPr

**Figure 4:**
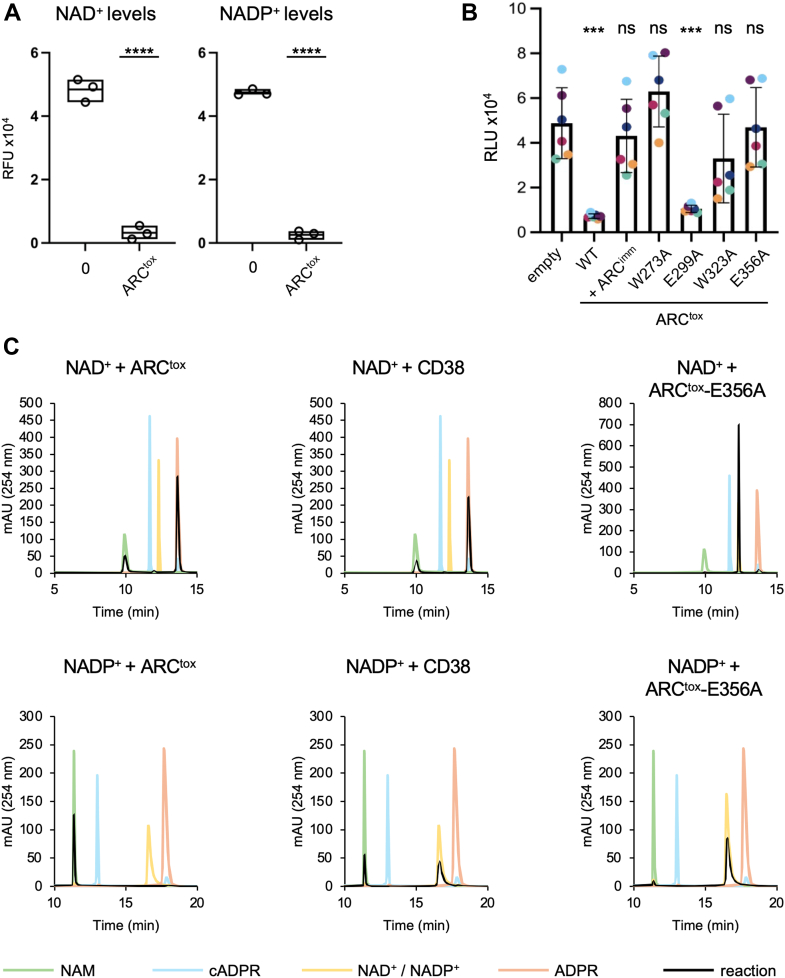
***P. ananatis* ARC^tox^ depletes NAD^+^ and NADP^+^ pools *in vivo* and *in vitro*.***A*, fluorescence-based assay showing NAD^+^ and NADP^+^ levels in untreated (0) and ARC^tox^-treated samples. NAD^+^ (*left panel*) or NADP^+^ (*right panel*) (0.6 mM) were incubated with the buffer (0) or with refolded ARC^tox^ (0.1 μM) *in vitro* for 30 min. RFU – relative fluorescence units. Boxes indicate standard deviation of the mean of three replicates. ∗∗∗∗ denotes a statistically significant difference between the two conditions, based on an unpaired *t*-test (*p* < 0.0001). *B*, luminescence assay measuring NAD^+^/NADH levels in *E. coli* DH5α cells producing the wild-type (WT) or the indicated mutated ARC^tox^ domains from the pNDM220 vector and ARC^imm^ from the pBAD33 vector. RLU – relative luminescence units. Bars show means ± SD from six independent experiments (same-color points represent replicates from the same experiment). ∗∗∗ denotes statistically significant difference compared to the control group (one-way ANOVA, *post hoc* Dunnett’s test, *p* < 0.001). ns = not significant. *C*, HPLC chromatograms of the products of the reactions of NAD^+^ (*upper panels*) and NADP^+^ (*lower panels*) in the presence of ARC^tox^ (*left panels*), CD38 (*middle panels*) or of the ARC^tox^ E356A variant (*right panels*) subjected to mixed-mode RP/anion-exchange Atlantis Premier BEH C18 AX column. The products are shown with the *black lines*. Chromatograms of selected standard analytes are shown in color (*green*, nicotinamide; *orange*, NAD^+^/NADP^+^; *flesh*, ADP-ribose; *blue*, cyclic ADP-ribose). HPLC analysis controls used to assess NAD^+^/NADP^+^ spontaneous hydrolysis and nucleotide contamination of ARC^tox^ preparations are presented in Figure S5.

We next assessed the enzymatic activity of the purified, refolded ARC^tox^ domain. *In vitro* assays showed efficient degradation of both NAD^+^ and NADP^+^ (Fig. 4*A*). NAD^+^ degradation activity was confirmed *in vivo* by measuring total NAD^+^ levels in *E. coli* cells after 1 h of induction of the ARC^tox^ domain: Figure 4*B* shows that expression of ARC^tox^ led to a drastic decrease of cellular NAD^+^ levels, confirming enzymatic activity in the cytosol. Co-expression of ARC^imm^ or expression of the ARC^tox^ catalytically inactive mutants restored NAD^+^ levels to near baseline, except for the E299A mutant, which retained a level of degradation comparable to that of the wild-type toxin.

To get further information on the reaction catalyzed by ARC^tox^, we performed high-pressure liquid chromatography (HPLC) to identify the reaction products. Upon incubation with ARC^tox^, NAD^+^ and NADP^+^ were converted into nicotinamide and ADP-ribose (Fig. 4*C*, left panels). No cADPr was, however, detected, likely due to its immediate hydrolysis to ADP-ribose (53). Consistent results were obtained with the well-characterized human CD38 ADP-ribosyl cyclase (Fig. 4*C*, middle panels), further supporting functional conservation. These products were specifically due to ARC^tox^ activity, as (1) mutation of the ARC^tox^ E356 catalytic residue prevented NAD^+^ and NADP^+^ hydrolysis (Fig. 4*C*, right panels), (2) NAD^+^ and NADP^+^ did not spontaneously hydrolyze during the assay (Fig. S5*A*), and (3) no nucleotide was detected by HPLC in the ARC^tox^ protein preparations (Fig. S5*B*).

### ARC domains are widespread and associated with polymorphic toxin systems

**Figure 5:**
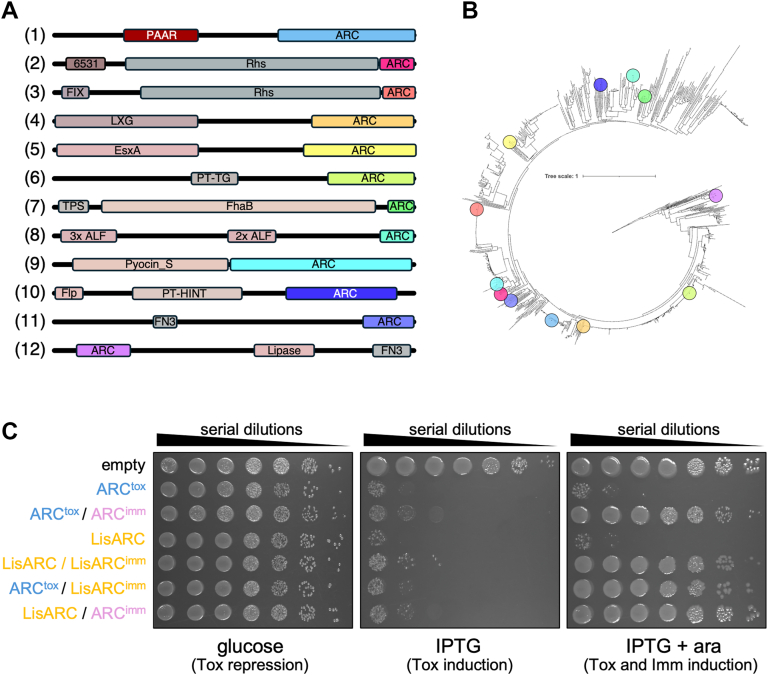
**Active ARC toxin domains are widespread across bacteria.***A*, domain architectures of selected proteins containing ARC domain homologs (1, *Pantoea ananatis* WP_013026797; 2, *Agrobacterium rubi* NTF28035; 3, *Burkholderia gladioli* WP_186165364; 4, *Listeria monocytogenes* WP_120135998; 5, *Gordonia jinhuaensis* WP_188589035; 6, *Lactiplantibacillus plantarum* WP_076633504; 7, *Stenotrophomonas maltophilia* OCK46403; 8, *Nonomuraea fuscirosea* WP_364663299; 9, *Marinobacter shengliensis* WP_138437386; 10, *Pendulispora albinea* WP_394825747; 11, *Allomuricauda sp.* RPG31737; 12, *Streptomyces mirabilis* WP_388533272). ARC domains are indicated in bright colors. Comparisons of the AlphaFold3 structural models of each of these ARC domains with *P. ananatis* ARC^tox^, and their statistics are shown in Figures S6 and S7, respectively. *B*, cladogram of 1075 homologs of *P. ananatis* ARC^tox^. The positions of the selected ARC domains shown in *panel A* are indicated by the same color code. *C*, toxicity assay in the heterologous host *E. coli.* Overnight cultures of *E. coli* cells expressing the indicated ARC domain (*P. ananatis* ARC^tox^ or *L.monocytogenes* ARC, LisARC) from the low-copy vector pNDM220, and the indicated immunity proteins (*P. ananatis* ARC^imm^ and *L. monocytogenes* LisARC^imm^) from the pBAD33 vector were serially diluted (10^-0^ to 10^-6^) and spotted on LB agar plates, supplemented with 1% of glucose (*left* panel, toxin repression), 0.02 mM of IPTG (*middle* panel, toxin induction), and 0.02 mM of IPTG and 1% of arabinose (*right* panel, toxin and immunity induction).

To explore the evolutionary context of ARC^tox^, we searched for homologous domains across bacterial genomes. ARC-like domains were found throughout the bacterial tree of life (Fig. 5, *A* and *B*), exclusively associated with secretion-targeting domains such as PAAR, RHS, FIX (T6SS), LXG and EsxA (T7SS), TPS (two-partner T5SS), and other extracellular or cell-wall anchoring elements like pyocins, Flp pilins, and fibronectin type III (Fn3) domains (54, 55, 56, 57) (Fig. 5*A*). Structural modeling showed that all these ARC homologs adopt the typical CD38-like ADP-ribosyl cyclase fold (Figs. S6 and S7).

Based on amino acid sequence identity (>70%), the closest homologues of *P. ananatis* ARC^tox^ are encoded within *Listeria* spp. genomes and systematically associated with T7SS-dependent LXG domains. Toxicity assays of a representative T7SS ARC domain from *Listeria monocytogenes* (LisARC and ARC^tox^ amino-acid identity/similarity: 68.6/82.5%) confirmed that it is toxic when expressed in *E. coli* and was neutralized by its cognate immunity protein (Fig. 5*C*). Further cross-protection experiments demonstrated that both *Pantoea* ARC^tox^ and LisARC are neutralized by non-cognate immunity proteins (Fig. 5*C*, LisARCimm and ARC^imm^ amino-acid identity/similarity: 38.2/67.1%). The high identity between ARC^tox^ and LisARC, and the cross-neutralization by their immunities suggest that ARC domains have been recently recruited to different secretion systems.

### ARC^tox^ can be secreted *via* a heterologous delivery system

**Figure 6:**
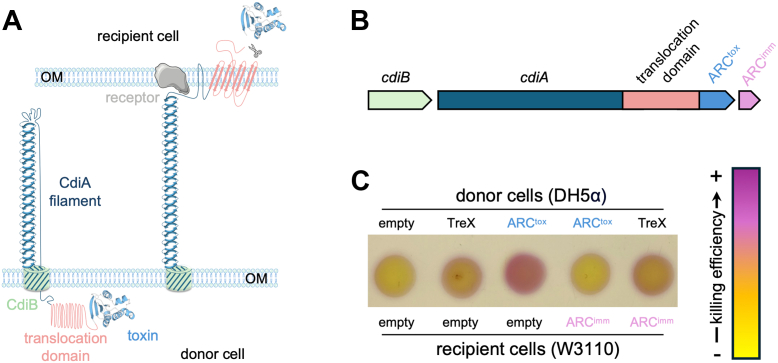
**CDI can be engineered to deliver ARC.***A*, schematic model of the mechanism of action of Contact-Dependent growth Inhibition (CDI) systems. The outer membrane component CdiB (*light green*) translocates CdiA (*dark blue*) to the donor cell surface. The receptor-binding domain of CdiA recognizes and binds to a specific receptor (*grey*) on the surface of the recipient cell. Then the translocon domain (*flesh*) delivers the toxin (*blue*) into the recipient cell. *B*, schematic representation of the CDI chimera engineered for ARC^tox^ delivery by the *E. coli* CdiAB CDI system. The *cdiB* and *cdiA* genes are shown in *light green* and *dark blue*, respectively. The grafted translocation domain and *P. ananatis* ARC^tox^ domain are shown in *flesh* and *blue*, respectively. ARC^imm^ is shown in *pink*. *C*, interbacterial competition assay. Donor (*E. coli* DH5α) and recipient (*E. coli* W3110) cells producing the indicated chimera CDIs (*P. ananatis* ARC^tox^ and *Xenorhabdus bovienii* ART, TreX) or ARC^imm^ were mixed at a 1:2 (v:v) ratio, spotted onto LB-agar, and incubated for 4 h at 37 °C. Recipient cell lysis after competition was revealed using CPRG (*yellow*), which turns purple upon hydrolysis by β-galactosidase from lysed *lacZ*^*+*^ recipient cells. The killing efficiency heatmap (from *yellow* (no killing) to *purple* (killing)) is shown on the *right*.

To test whether ARC^tox^ could be redirected through another delivery system, we engineered a chimera using the *E. coli* CdiAB contact-dependent inhibition (CDI) system, as done recently with the *Xenorhabdus bovienii* TreX ADP-ribosyl transferase (58). For this, ARC^tox^ was fused to the C-terminal CdiA stick domain, after the conserved VENN motif that delimitates CdiA transport and toxic domains (Fig. 6, *A* and *B*). Exposition of this chimeric effector to the *E. coli* DH5α cell surface was toxic to *E. coli* W3110 competitor cells but not to cells expressing the ARC immunity protein, demonstrating effective delivery and functional toxin activity (Fig. 6*C*). In contrast, ARC^imm^ did not protect against an unrelated CDI toxin (*Xb*ART), indicating specific poisoning of recipient cells with ARC^tox^ (Fig. 6*C*).

## Discussion

In this article, we report the characterization of a novel and conserved class of versatile polymorphic toxin domains, ARC^tox^, sharing structural and functional characteristics of the human CD38 ADP-ribosyl cyclase. We demonstrate that the C-terminal extension of a specialized PAAR protein from *P. ananatis*, PANA_2924, acts as a potent cytotoxic effector, exhibiting both antibacterial and anti-eukaryotic activity through depletion of the cellular NAD^+^/NADP^+^ pool. This toxin is neutralized by a tightly binding immunity protein (PANA_2923), forming a canonical toxin–immunity module reminiscent of those employed in interbacterial conflict. Very recently, the ARC antibacterial Tac1 toxin associated with the *Streptoccocus parasanguinis* T7SS was functionally and structurally characterized, demonstrating that it hydrolyzes NAD^+^ and NADP^+^ into ADP-ribose and ADP-ribose-2′-phosphate, respectively (59).

Remarkably, structural and biochemical data indicate that ARC^tox^ and Tac1 are bacterial analogs of human CD38, a multifunctional enzyme involved in immune signaling and NAD metabolism (49, 50, 59), revealing an unexpected link between polymorphic bacterial effectors and eukaryotic cell biology. The discovery of this relationship between ARC^tox^ and Tac1, and CD38 is significant both structurally and functionally. The ARC toxins adopt a globular fold closely resembling CD38, characterized by a β-sheet core capped by α-helices. DALI analysis and high-resolution crystal structures reveal that these enzymes share not only overall architecture but also precise placement of key catalytic residues—two glutamates and two tryptophans—which define the active site of the CD38 family (36, 51, 52). Mutational inactivation of these residues in ARC^tox^ completely abrogates its toxic activity, confirming their critical role in catalysis. Importantly, this enzymatic function is not merely structural mimicry: ARC^tox^ directly hydrolyzes NAD^+^ and NADP ^+^ into ADP-ribose and nicotinamide, recapitulating the enzymatic outcome of CD38 activity in eukaryotic cells (36). The absence of cyclic ADP-ribose (cADPr), likely due to its rapid hydrolysis, aligns with the catalytic profile of some CD38-like enzymes that favor hydrolysis over cyclization (53). These data also suggest that ARC^tox^ antibacterial toxicity is not due to the accumulation of cADPr, which could impact undefined signaling pathways in bacteria, but rather poisons target cells by depleting the pool of NAD^+^/NADP^+^. Indeed, the recent work of Colautti *et al.* demonstrated that the *S. parasanguinis* T7SS Tac1 ARC toxin hydrolyzes NAD^+^ with a rate of 12,000 molecules/min (59), which is comparable with previous NAD glycohydrolase toxins (60, 61). The ability of a bacterial toxin to deplete NAD^+^, a central metabolic cofactor, highlights a sophisticated mechanism of intercellular antagonism that targets core bioenergetic processes. While many known bacterial toxins act by degrading nucleic acids, lipids or proteins (14, 16, 19), ARC^tox^ represents a distinct class of metabolic toxins, sabotaging host or competitor metabolism through NAD^+^ depletion. Other toxins belonging to the ART-fold superfamily have been shown to utilize the NAD^+^ or NADP^+^ for ADP-ribosylation of protein or of RNA targets, or for NAD^+^ hydrolysis (60, 61, 62, 63, 64, 65, 66, 67, 68). This ART-fold superfamily comprises a conserved core fold corresponding to a central split β-sheet with a conserved order of strands (69). The ARC fold is highly distinct, but our genomic analysis showed that it is also highly widespread, but previously only reported in eukaryotic CD38. In any case, the NAD^+^ depletion strategy likely confers a competitive advantage in polymicrobial environments, especially in the plant rhizosphere or during host colonization, where nutrient access and niche competition are intense (2, 3, 70). The dual activity of ARC^tox^ against both bacterial and eukaryotic cells further suggests that this effector may function during both interbacterial competition and plant–pathogen interactions, consistent with the known virulence roles of *P. ananatis* T6SS in onion infection (37, 38).

The conservation between ARC^tox^ and CD38 extends beyond structure and catalysis; it raises fascinating evolutionary questions. The phylogenetic distribution of ARC domains across diverse bacterial lineages, and their consistent association with modular toxin architectures (PAAR, RHS, LXG, TPS, Fn3, *etc.*), suggests that these domains have been evolutionarily co-opted for secretion *via* multiple delivery systems, including T6SS, T5SS, T7SS, and CDI, or for cell surface exposition. This modularity, which is a hallmark of polymorphic toxins, enables effector domains to be flexibly redeployed across different antagonistic contexts. Yet, the fact that the same enzymatic fold underlies both a eukaryotic signaling enzyme and a prokaryotic toxin raises the possibility of an ancient common ancestry or repeated events of horizontal gene transfer followed by domain repurposing. An additional possibility is that the similarity between ARC^tox^ and CD38 arose through convergent evolution, with both proteins independently evolving similar fold and catalytic mechanism due to functional constraints on NAD^+^ metabolism. The metabolic versatility of the CD38/ARC fold, capable of catalyzing both cyclization and hydrolysis reactions on NAD^+^ substrates, may have facilitated its functional diversification across kingdoms.

Our findings also highlight the high adaptability of bacterial effector systems. The incorporation of ARC^tox^ into a heterologous CDI system, where it successfully mediates contact-dependent killing of target cells, highlights the modularity and portability of these effectors. This property, combined with the cross-reactivity of immunity proteins (*i.e.*, cross-protection between *Pantoea* and *Listeria* ARC^tox^ modules), suggests that ARC^tox^ domains can be rationally engineered for synthetic biology applications, such as programmable antibacterial agents, targeted delivery systems, or novel biocontrol strategies in agriculture. Moreover, the cross-protection between non-cognate immunity proteins may reflect conserved structural features of the catalytic cleft, hinting at convergent evolution or selective pressure to maintain compatibility within toxin-immunity networks.

The biological implications of ARC^tox^ extend to host-pathogen interactions as well. Given its demonstrated anti-eukaryotic activity in yeast, one may hypothesize that ARC^tox^-like proteins are deployed during plant infection, where they could modulate host immunity, disrupt cellular metabolism, or interfere with NAD^+^-dependent signaling pathways. Such roles would mirror the function of CD38 in immune cells, albeit in a pathogenic rather than physiological context. Further investigations into the role of ARC^tox^ in plant–microbe interactions, including its potential delivery into plant cells or modulation of host NAD^+^ pools, could reveal novel mechanisms of bacterial virulence.

Our broader genomic survey of ARC domain distribution indicates that this ARC NAD hydrolase toxin architecture is widespread across the bacterial tree of life. Its association with diverse secretion and anchoring domains supports the notion that ARC^tox^ is a core component of the bacterial competitive arsenal. The presence of ARC domains in systems as distinct as pyocins and T7SS suggests that ARC^tox^ may contribute to antagonism in a variety of ecological contexts, from soil and plant microbiomes to animal hosts and clinical infections.

## Experimental procedures

### Strains, media and growth conditions

*E. coli* strain DH5α was used for cloning, toxicity tests, and NAD/NADH-Glo Assay (Promega). *E. coli* BL21 (DE3) and W3110 strains were used for protein production and bacterial competition, respectively. *E. coli* cells were grown at 37 °C in Lysogeny Broth (LB) with agitation or on LB agar (1.5%) plates. When needed, media were supplemented with ampicillin (50–100 μg.mL^-1^), chloramphenicol (30 μg.mL^-1^), kanamycin (50 μg.mL^-1^), or streptomycin (100 μg.mL^-1^). Gene expression was induced by the addition of 1% of L-arabinose, 50 to 100 μM of IPTG or repressed with 1% of glucose. *S. cerevisiae* strain W303 was used for toxicity assays. *S. cerevisiae* cells were grown at 30 °C in YPAD (Yeast extract Peptone Adenine Dextrose) or SD (Synthetic Dextrose minimal) media with agitation, or on YPAD, SD, or SG (Synthetic Galactose minimal) agar plates.

### Plasmid construction and mutagenesis

All cloning procedures were performed by restriction–ligation or restriction-free based methods. Oligonucleotides were purchased from IDT. DNA fragments encoding ARC^tox^ and ARC^imm^ were amplified from *P. ananatis* LMG 20103 using the Q5 polymerase (NEB). Vector backbones and full plasmid templates were amplified using PrimeSTAR Max DNA polymerase (Takara). DNA fragment encoding the *L. monocytogenes* LS1292 LXG ARC domain (LisARC) and its cognate immunity (LisARC^imm^) was synthesized by Twist Bioscience. LisARC and LisARC^imm^ fragments were then PCR-amplified using the Q5 polymerase. PCR products were purified using NucleoSpin Gel and PCR Clean-up columns (Macherey-Nagel), digested with the appropriate restriction enzymes (NEB), and repurified prior to ligation. Ligated plasmids were transformed into *E. coli* DH5α and screened by colony PCR using EconoTaq PLUS GREEN 2x Master Mix (Biosearch Technologies). Plasmid DNA was extracted using either the Wizard Plus SV Minipreps kit (Promega) or the NucleoSpin Plasmid kit (Macherey-Nagel) and verified by Sanger sequencing (Eurofins). Constructs were selected on LB agar plates containing ampicillin (for pRS416_Gal1, pNDM220, and pET-Duet1), chloramphenicol (for pBAD33), kanamycin (for pRSF-Duet1), or streptomycin (for pCDF-Duet1). Glucose (1%) was added for selection with the pNDM220 vector. Note that, despite several attempts, we never succeeded to clone the wild-type sequence of PANA_2924 C-terminal domain into pRS416_Gal1, probably due to its high toxicity and promoter leakiness in *E. coli*. However, we obtained an attenuated mutant bearing a 1 bp (A) deletion at position 435. Toxin and immunity operon were cloned into pET-Duet1, while immunity genes were also cloned individually into pBAD33, pRSF-Duet1, or pCDF-Duet1. Site-directed mutagenesis of ARC^tox^ catalytic residues was performed using QuickChange mutagenesis by PCR-amplifying the whole plasmid using primers bearing the desired mutations and the pNDM220-ARC^tox^ plasmid as template. PCR products were cleaned up from gels to eliminate the template plasmid, then phosphorylated and ligated (T4 DNA ligase, NEB) prior to transformation. The pCH10163-ARC^tox^-ARC^imm^ plasmid, encoding ARC^tox^ fused to *E. coli* CdiA was generated by restriction-free cloning, using overlapping regions to fuse the desired fragments.

### Toxicity assays in *E. coli* and yeast

*E. coli* DH5α cells were co-transformed with the pNDM220 and pBAD33 plasmid pairs bearing either ARC^tox^, ARC^tox^ mutants or LisARC and ARC^imm^ or LisARC^imm^, respectively, or empty vectors as controls. Transformants were selected on LB agar plates supplemented with ampicillin, chloramphenicol, and 1% of glucose. Overnight cultures grown in the presence of antibiotics and glucose were diluted in 10-fold series, and 10-μl drops were spotted on LB agar plates containing antibiotics and either 1% glucose (repression conditions) or 1% arabinose (immunity induction from pBAD33) with 0.05 mM or 0.02 mM IPTG (toxin induction from pNDM220). Plates were incubated at 37 °C for 16 to 20 h before imaging.

*S. cerevisiae* strain W303 was transformed with the pRS416_Gal1 encoding ARC^tox^ or an empty vector as control and selected on SD agar plates. Overnight cultures grown in SD liquid medium were diluted in 10-fold series, and 5-μl drops were spotted onto SD (repression condition) and SG (induction condition) agar plates. Plates were incubated at 30 °C for 44 to 48 h before imaging.

### Purification of ARC^tox^-ARC^imm^ complex and ARC^tox^

Freshly transformed *E. coli* BL21 (DE3) cells carrying pET-hisTEV-ARC^tox^-ARC^imm^, pRSF-ARC^imm^, and pCDF-ARC^imm^ or pET-hisTEV-ARC^tox^ -E356A were grown overnight in LB medium supplemented with appropriate antibiotics. Cultures were diluted 1:100 into 2 L of LB and grown at 37 °C to an OD_600_ of 0.8. After addition of 0.5 mM of IPTG, cells were grown overnight at 16 °C with agitation. Cells were collected by centrifugation at 4000*g* and resuspended in lysis buffer (50 mM Tris-HCl pH 8.5, 250 mM NaCl, 1 mM TCEP) containing cOmplete protease inhibitor cocktail (Sigma-Aldrich). Cells were lysed by sonication, and the cell lysate was clarified by centrifugation at 20,000*g* for 45 min. The supernatant was filtered through a 0.45 μm filter and incubated with 2 ml TALON Metal Affinity Resin (Takara) pre-equilibrated with lysis buffer for 1 h at 4 °C with gentle mixing. The resin was washed five times with 15 bed volumes of lysis buffer, and bound proteins were eluted with 1 ml of elution buffer (50 mM Tris-HCl pH 8.5, 250 mM NaCl, 1 mM TCEP, 200 mM imidazole). For purification of ARC toxin alone, after the washing steps with lysis buffer, the resin was incubated with 8 M urea for 2 min, followed by one additional wash with 8 M urea. Refolding was performed by sequential washes with refolding buffer 1 (50 mM Tris-HCl, pH 8.5, 125 mM NaCl, 5% glycerol), followed by two washes with refolding buffer 2 (50 mM Tris-HCl pH 8.5, 250 mM NaCl, 1% glycerol). The toxin was then eluted using 1 ml of elution buffer. The His-TEV tag was removed from both the ARC^tox^/ARC^imm^ complex and ARC^tox^ by incubation with the purified TEV protease (1:100 M ratio of TEV protease:His-tagged protein) overnight at 4 °C. Proteins were further purified by size-exclusion chromatography (SEC) using a Superdex 75 Increase 10/300 Gl column (Cytiva) equilibrated in buffer A (20 mM Tris-HCl pH 8.5, 150 mM NaCl, 1 mM TCEP) for the ARC^tox^/ARC^imm^ complex, or buffer B (20 mM Tris-HCl pH 8.5, 150 mM NaCl) for ARC^tox^. The SEC column was calibrated using Gel filtration Standard (Biorad), which includes bovine thyroglobulin (670 kDa), bovine γ-globulin (158 kDa), chicken ovalbumin (44 kDa), horse myoglobin (17 kDa), and vitamin B_12_ (1.35 kDa) (Fig. S1).

### Crystallization, structure determination, and refinement of ARC^tox^

Following purification, tag removal, and gel filtration, ARC^tox^ toxin was concentrated to 13.4 mg mL^-1^ and subjected to crystallization screening using the sitting-drop vapor diffusion method at 20 °C. Crystallization drops were set up in Swissci 96-well 2-drop MRC plates (Molecular Dimensions) by mixing 0.5 μl of protein solution with 0.5 μl of reservoir solution. Drops were equilibrated against 70 μl of crystallization screens, including Crystal Screen I and II (Hampton Research), LMB, PACT Premier, JCSG+, and MemGold (Molecular Dimensions).

Diffraction-quality crystals of ARC^tox^ were obtained using a reservoir solution containing 0.2 M ammonium chloride, 0.1 M sodium acetate (pH 5.0), and 20% (w/v) PEG 6000. Prior to cryo-cooling in a liquid nitrogen stream, crystals were cryo-protected by briefly transferring them into reservoir solution supplemented with 20% glycerol. X-ray diffraction data were collected at the PROXIMA-2A (PX2A) beamline at the SOLEIL Synchrotron (Gif-sur-Yvette, France), integrated with XDS (71) and scaled with AIMLESS (72) from the CCP4 program suite (73). The structure was solved by molecular replacement using an AlphaFold3 model of ARC^tox^ as a search probe using PHASER (74) in PHENIX (75), with two molecules in the asymmetric unit. The initial map was excellent, and the model was manually improved in COOT (76) and subsequently refined with PHENIX (75). The final model was refined to a R_work_/R_free_ of 0.18/0.21 with excellent statistics (Table S1). The coordinates and structure factors were deposited in the Protein Data Bank (PDB) with accession code 9RO8.

### *In vitro* NAD(P)^+^ hydrolysis assay

Reactions were performed in 100 μl of PBS supplemented with 0.6 mM of NAD^+^ or of NADP^+^ (Roche). Purified, refolded ARC^tox^ was added to a final concentration of 0.1 μM. Samples were incubated at room temperature for 30 min. The reaction was then quenched by adding 50 μl of 6 M NaOH. Fluorescence was measured using a TECAN plate reader (excitation: 340 nm; emission: 420 nm) after 30 min of incubation at room temperature in the dark.

### *In vivo* NAD/NADH-Glo assay

*E. coli* strain DH5α cells producing wild-type and mutant ARC^tox^ domains, or both ARC^tox^ and ARC^imm^ were grown in medium supplemented with antibiotics and 1% glucose at 37 °C with agitation until OD_600_ reached 0.5. Cells were then washed with LB medium and resuspended in fresh LB supplemented with 0.05 mM of IPTG, 1% of arabinose, and antibiotics. After incubation for 1 h at 37 °C with agitation, 10^9^ cells were mixed with the NAD/NADH-Glo detection reagent (Promega), and the measure of NAD^+^ levels was done according to the manufacturer’s instructions.

### Analysis of ARC^tox^ activity by high pressure liquid chromatography

NAD^+^ or NADP^+^ (1.5 mM) were incubated for 30 min with 0.1 μM of wild-type or E356A variant ARC^tox^ or of purified CD38 soluble domain (kind gift of Alain Roussel, LISM, Marseille, France), or with buffer alone. As further control, 0.1 μM of ARC^tox^ was incubated with buffer alone to ensure no contamination of ARC^tox^ preparations with nucleotides. Proteins were then retained onto Amicon Ultra 3 kDa filters (Sigma-Aldrich) by centrifugation at 11,000*g* for 15 min. The flow-through was injected into an Agilent 1260 Infinity HPLC system equipped with a mixed-mode RP/anion-exchange Atlantis PREMIER BEH C_18_ AX column. Analytes were separated at a flow rate of 1 mL min^-1^ for 40 min, with a gradient profile of 100% Solvant A (10 mM ammonium formate pH3.0 for NADP^+^, 20 mM ammonium formate pH2.9 for NAD^+^) for 5 min, linear gradient to 50% Solvant A/50% Solvent B (acetonitrile) for 30 min, and 100% Solvent B for 5 min. Analytes were detected by absorbance at 254 nm. The retention times were compared to standards run on the same column (Nicotinamide (NAM) 250–450 μM, NAD^+^ 40 μM, NADP^+^ 150 μM, ADP-ribose 150–200 μM, cyclic ADP-ribose 60–160 μM).

### Bacterial competition assay

Interbacterial competition was measured using the LAGA assay (77). This assay is based on the degradation of chlorophenol-red β-D-galactopyranoside (CPRG), a membrane-impermeable chromogenic substrate of the β -galactosidase, which is cleaved by the β -galactosidase released by the recipient cell lysis. *E. coli* DH5α cells producing CdiB and the CdiA chimera proteins were used as donors, whereas *E. coli* W3110 (*lacZ*^+^) cells were used as recipient. Both strains were grown separately to an OD_600_ of 0.5, washed twice with LB medium supplemented with 0.05 mM IPTG, and mixed to a 1:2 (donor:recipient) ratio. 10 μl of the mixtures were spotted onto LB agar plates supplemented with 0.05 mM of IPTG. Plates were incubated for 4 h at 37 °C, and recipient cell lysis was observed by the addition of a 10-μl drop of 2 mM of CPRG (Roche) on the top of each spot.

### Statistical analysis

*In vitro* ARC^tox^ activity data were analyzed using an unpaired *t*-test. *In vivo* data from NAD/NADH-Glo assay were analyzed using one-way ANOVA followed by Dunnett’s *post hoc* test. All analyses were performed using the GraphPad Prism software.

## Data availability

The *P. ananatis* PANA_2924 ARC^tox^ structure has been deposited to the Protein Data Bank (PDB) under accession code 9RO8.

## Supporting information

This article contains supporting information (seven Supplemental Figures and four Supplemental Tables) (23, 58, 78, 79, 80).

## Conflict of interest

The authors declare that they do not have any conflicts of interest with the content of this article.
