# Supplementary material for: Widespread deployment of the human CD38 ADP-ribosyl cyclase fold in antibacterial and anti-eukaryotic polymorphic toxins
Source: J Biol Chem. 2025 Sep 27;301(11):110775. doi: 10.1016/j.jbc.2025.110775 (PMC12605009; doi:10.1016/j.jbc.2025.110775)
Supplement: Supplemental Figures [file mmc1.pdf]

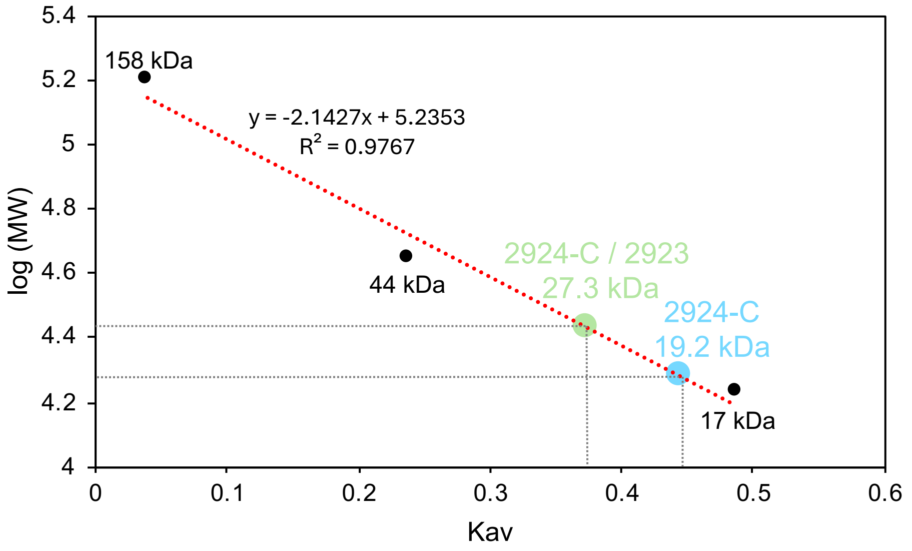


**Supplemental Figure S1. SEC standard curve and experimental molecular weight determination.** A standard curve (red dotted line) was established using Bio-Rad molecular weight standards, including bovine thyroglobulin (670 kDa), bovine γ-globulin (158 kDa), chicken ovalbumin (44 kDa), horse myoglobin (17 kDa), and vitamin $B_{12}$ (1.35 kDa). Because bovine thyroglobin and vitamin $B_{12}$ fall outside the range of the Superdex 75 column, they were not used to establish the standard curve. The partition coefficient $K_{av}$ was calculated according to the equation $K_{av} = (V_e - V_0) / (V_C - V_0)$, where $V_e$ is the elution volume of the analyte, $V_c$ is the column bed volume (determined from vitamin $B_{12}$ elution), and $V_0$ is the void volume (determined from thyroglobulin elution). The linear regression equation and corresponding $R^2$ value are shown in the plot. MW, molecular weight.

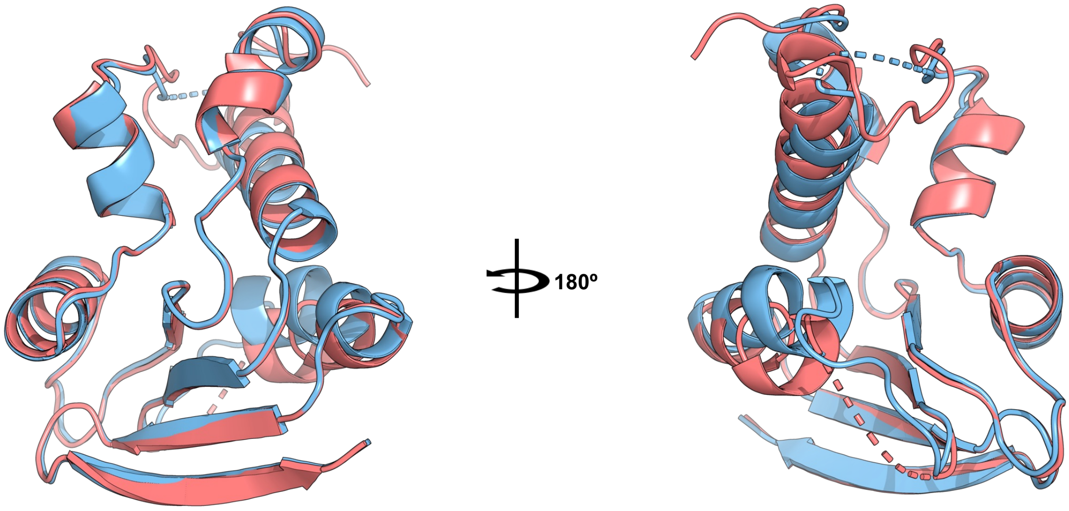


**Supplemental Figure S2. Comparison of the crystal structures of the two chains of the PANA_2924 C-terminal extension asymmetric unit.** Superimposition of the crystal structures of chains A (blue) and B (red) of the PANA_2924 C-terminal domain. The dotted lines highlight the positions of the loops that are missing in one structure but visible in the other.

A

```
                W273
PANA_2924  KSLINLKEIEP--QLATDP---DSAFFWSGRTEGVGGPDVAEAIAK-SRG
               ..::.:|  :|.|..   :....|| |.:     |:|....: .|.
CD38       ----TEEDYQPLMKLGTQTVPCNKILLWS-RIK-----DLAHQFTQVQRD
                W125

           E299                                        W323
PANA_2924  GVTLESTI-----------------KDKNIKMPEWDFD---NPQSIKAWE
           ..|||.|:                 |......|:|..|   ||.|: .|:
CD38       MFTLEDTLLGYLADDLTWCGEFDTSKINYQSCPDWRKDCSNNPVSV-FWK
           E146                                        W189

                                        E356
PANA_2924  DVSASYAKQVSGEVRAVVGQS----LREGNIWENVELPRLMGNDNVTKIT
           .||..:|:.....|..::..|    ..:.:.:.:||:..|...    |:.
CD38       TVSRRFAEAACDVVHVMLDGSRSKIFDKDSTFGSVEVHNLQPE----KVQ
                                        E226

PANA_2924  TI-----------------DP-LSQTEKVIFVRDN--------
           |:                 || :.:.|.:|..|:.
CD38       TLEAWVIHGGREDSRDLCQDPTIKELESIISKRNIQFSCKNIY
```

B

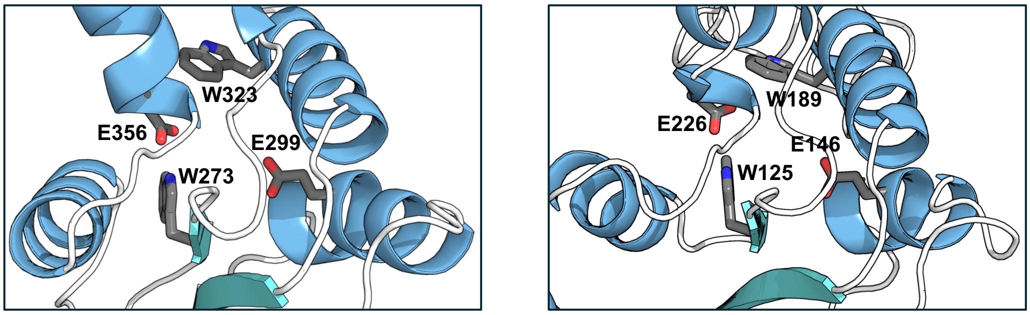


2924-C (PDB: 9RO8)
ARC$^{tox}$

CD38 (PDB: 2PGJ)

**Supplemental Figure S3. Comparison of the amino acid sequences and crystal structures of the PANA_2924 C-terminal extension (PDB: 9RO8) and human CD38 (PDB: 2PGJ). (A)** Sequence alignment of PANA_2924 and CD38 C-terminal domains, highlighting catalytic residues. PANA_2924 and CD38 catalytic residues are shown in blue and red, respectively. The alignment was generated using the EMBOSS Needle pairwise alignment tool. **(B)** Structural arrangement of conserved catalytic residues (shown in grey) within the $NAD^+$/$NADP^+$ binding pockets of the PANA_2924 C-terminal extension (left) and CD38 (right).

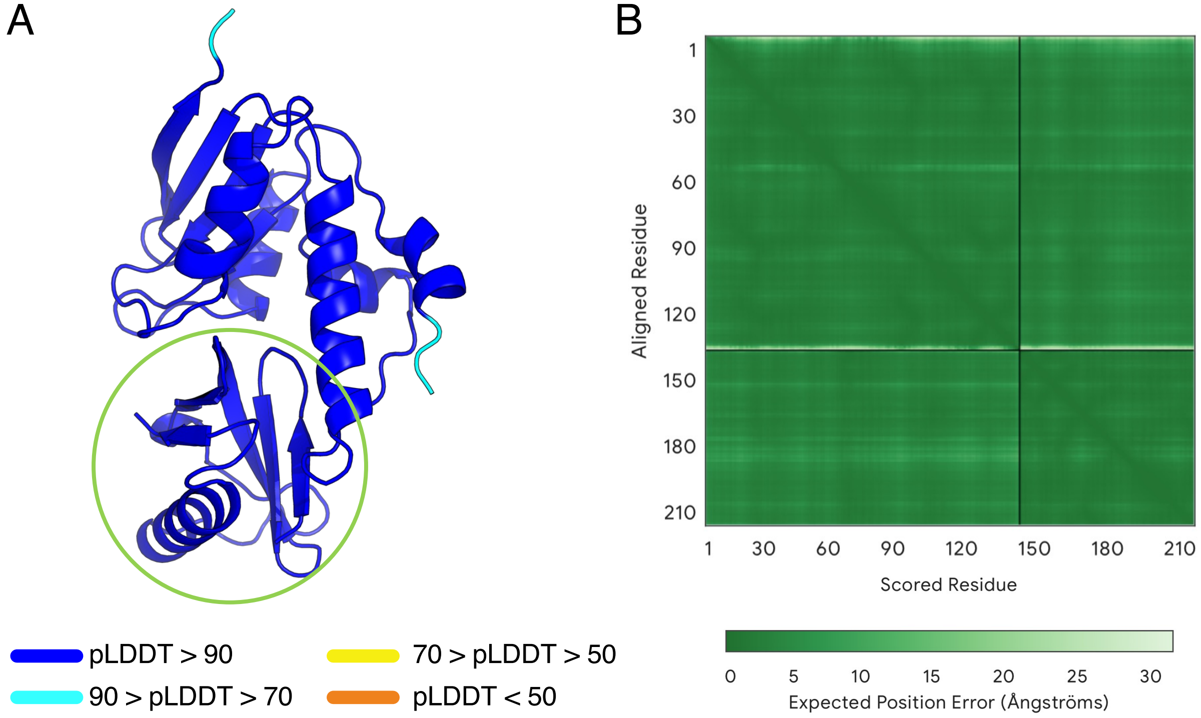


**Supplemental Figure S4. Confidence scores of the AlphaFold3 structural model of the ARC$^{tox}$/ARC$^{imm}$ complex. (A)** Structural model of the ARC$^{tox}$-ARC$^{imm}$ complex colored by predicted Local Distance Difference Test (pLDDT) scores, ranging from blue (high confidence, >90) to orange (low confidence, <50). The green circle indicates ARC$^{imm}$. **(B)** Predicted Aligned Error (PAE) plot showing the expected positional error (in Å) for each residue pair, with dark green indicating high confidence and lighter green indicating lower confidence.

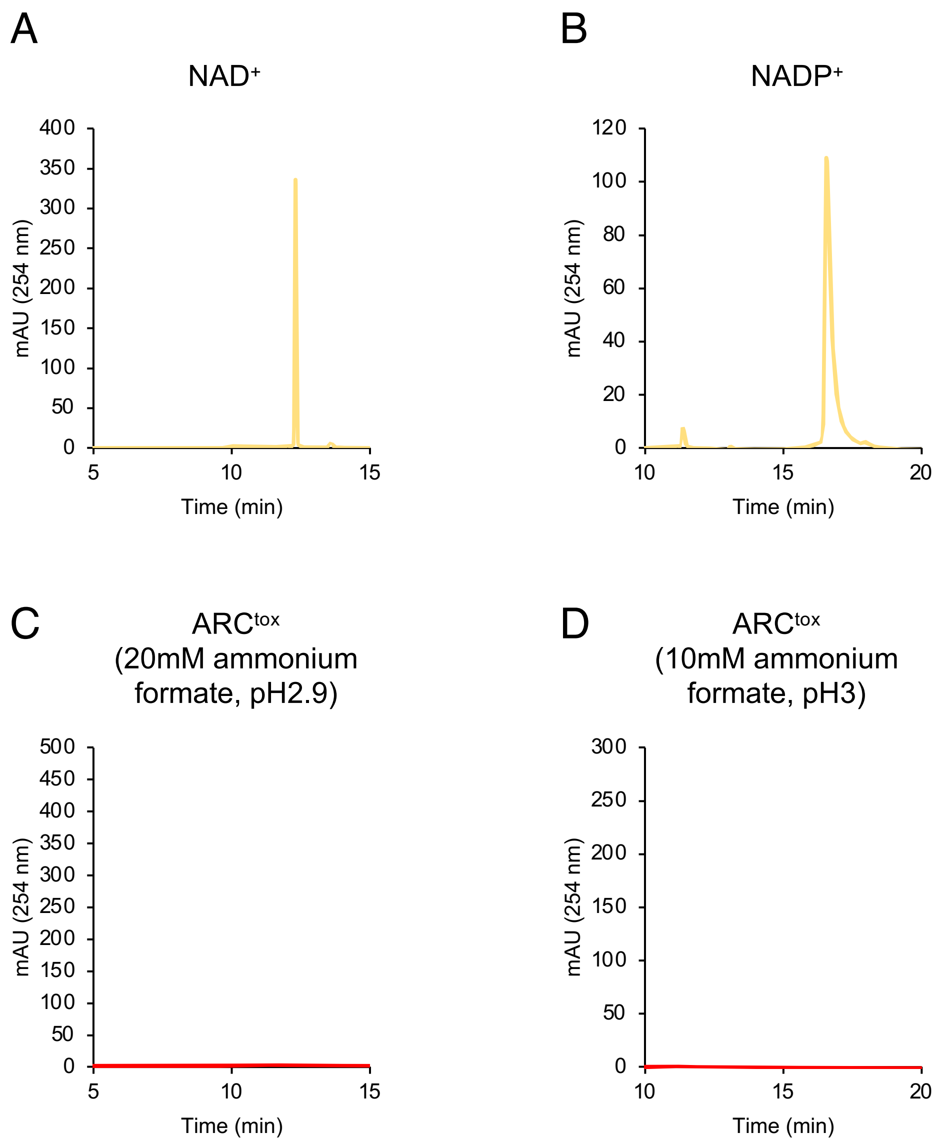


**Supplemental Figure S5. Control chromatograms.** Mixed-mode HPLC chromatograms of the products of the reactions of $NAD^+$ (**A**) and $NADP^+$ (**B**) in the presence of buffer (20 mM ammonium formate, pH2.9, for $NAD^+$ or 10 mM ammonium formate, pH3, for $NADP^+$), as well as for the $ARC^{tox}$ alone in the presence of ammonium formate buffers (**C** and **D**). Note that a weak peak corresponding to nicotinamide is always visible for $NADP^+$ alone.

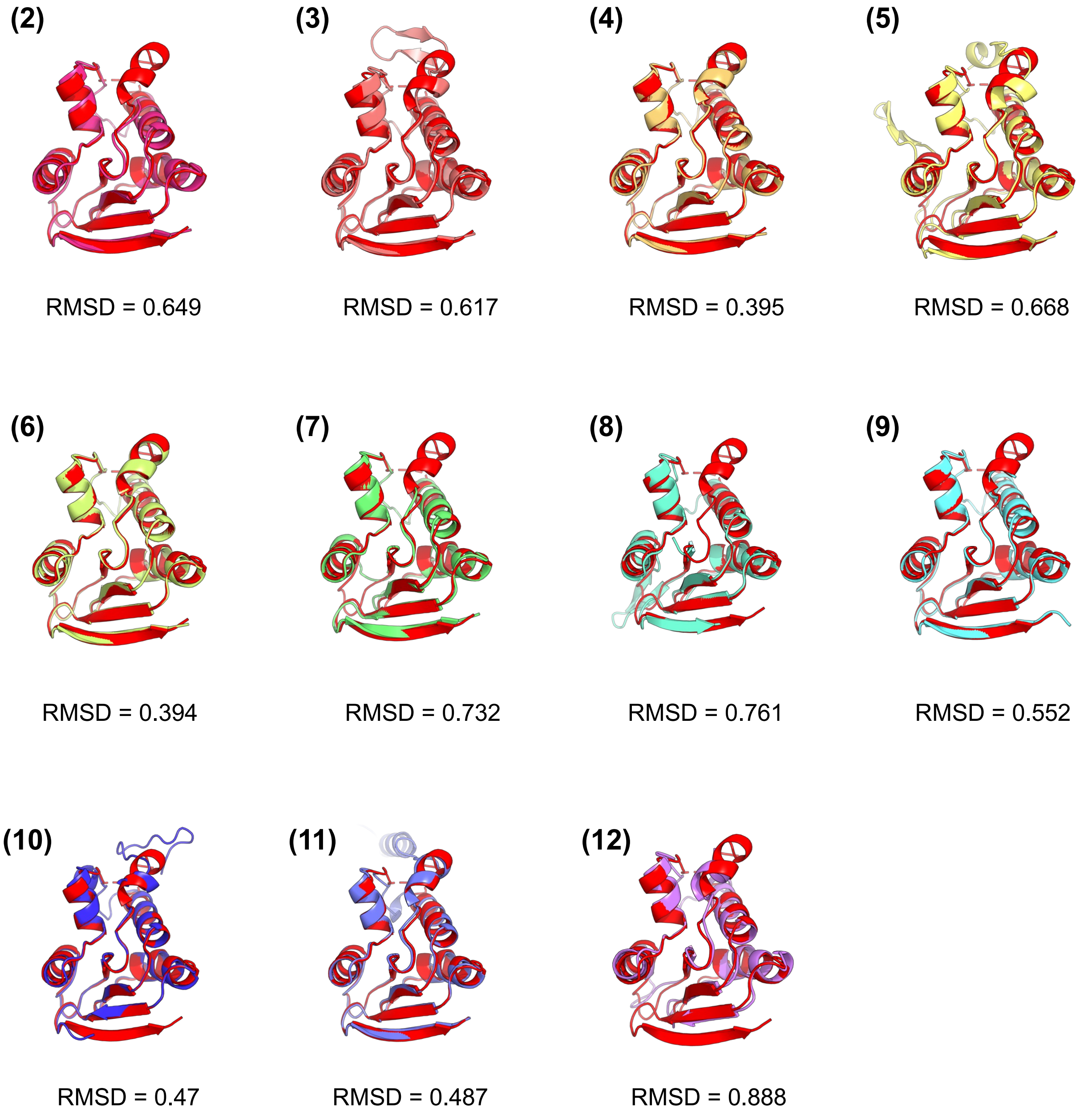


**Supplemental Figure S6. Alignment of the *P. ananatis* ARC$^{tox}$ crystal structure (red) with AlphaFold3 structural models of ARC domains.** 2, *Agrobacterium rubi* NTF28035; 3, *Burkholderia gladioli* WP_186165364; 4, *Listeria monocytogenes* WP_120135998; 5, *Gordonia jinhuaensis* WP_188589035; 6, *Lactiplantibacillus plantarum* WP_076633504; 7, *Stenotrophomonas maltophilia* OCK46403; 8, *Nonomuraea fuscirosea* WP_364663299; 9, *Marinobacter shengliensis* WP_138437386; 10, *Pendulispora albinea* WP_394825747; 11, *Allomuricauda sp.* RPG31737; 12, *Streptomyces mirabilis* WP_388533272. Root-Mean-Square Deviation (RMSD) values are indicated below each superimposition. The pLDDT scores for AlphaFold3 structural model for each ARC domain are shown in Supplemental Figure S7.

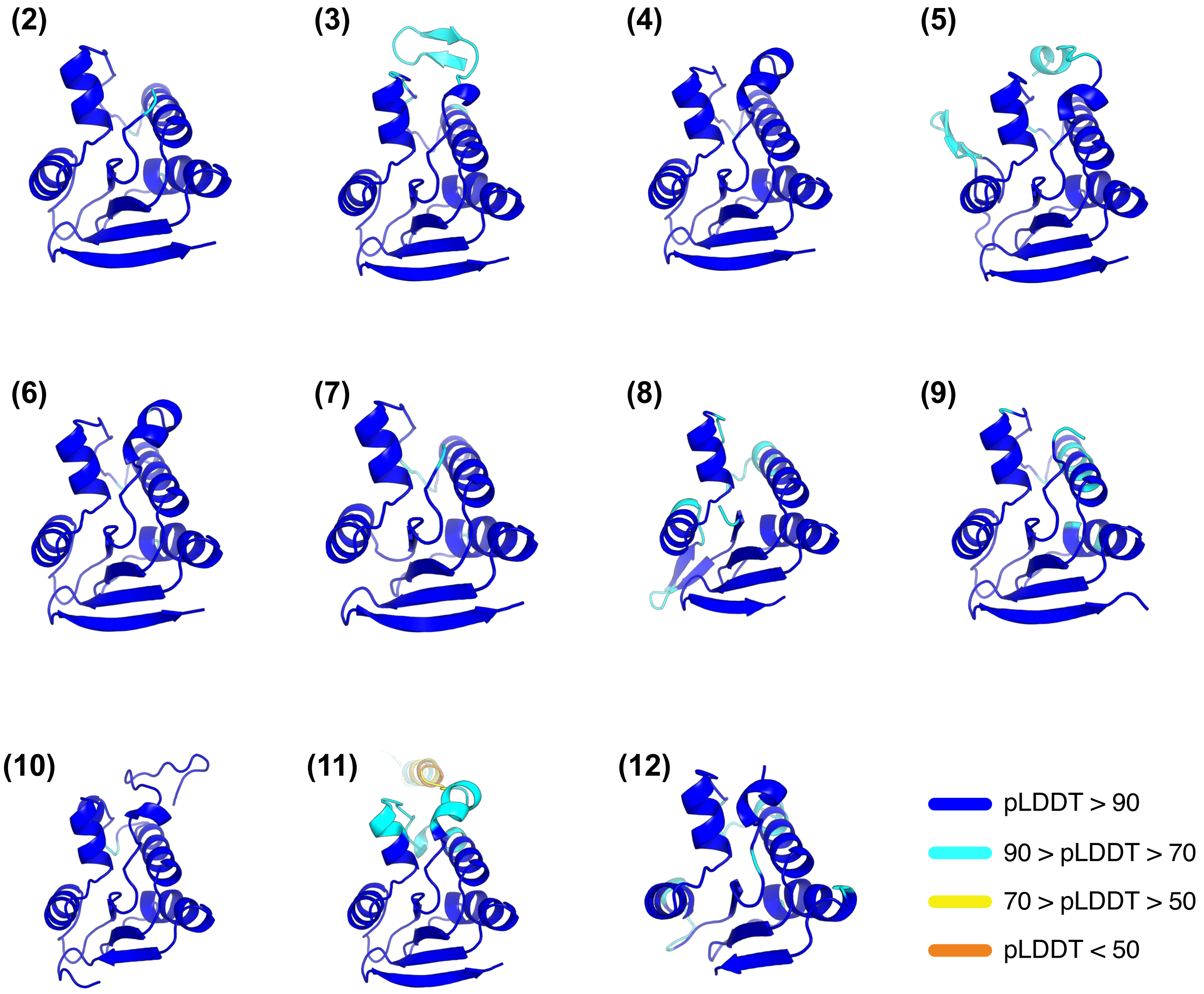


**Supplemental Figure S7. AlphaFold3 structural models of ARC domains** colored by pLDDT scores, ranging from blue (high confidence, >90) to orange (low confidence, <50). 2, *Agrobacterium rubi* NTF28035; 3, *Burkholderia gladioli* WP_186165364; 4, *Listeria monocytogenes* WP_120135998; 5, *Gordonia jinhuaensis* WP_188589035; 6, *Lactiplantibacillus plantarum* WP_076633504; 7, *Stenotrophomonas maltophilia* OCK46403; 8, *Nonomuraea fuscirosea* WP_364663299; 9, *Marinobacter shengliensis* WP_138437386; 10, *Pendulispora albinea* WP_394825747; 11, *Allomuricauda sp.* RPG31737; 12, *Streptomyces mirabilis* WP_388533272.
