# Supplementary material for: Widespread deployment of the human CD38 ADP-ribosyl cyclase fold in antibacterial and anti-eukaryotic polymorphic toxins
Source: J Biol Chem. 2025 Sep 27;301(11):110775. doi: 10.1016/j.jbc.2025.110775 (PMC12605009; doi:10.1016/j.jbc.2025.110775)
Supplement: Supplemental Tables [file mmc2.pdf]

Supplemental Table 1. Data collection and refinement statistics.

	**ARC^{tox} (PDB : 9RO8)**
**Data collection**	
**Resolution range**	39.66 - 1.594 (1.63 - 1.59)
**Space group**	P 21 21 21
**Unit cell**	44.23 70.213 96.119 90 90 90
**Total reflections**	
**Unique reflections**	40577 (2746)
**Multiplicity**	
**Completeness (%)**	99.73 (96.38)
**Mean I/sigma(I)**	17.0 (2.2)
**Wilson B-factor**	21.48
**R-merge**	0.092 (1.053)
**R-meas**	0.096 (1.099)
**R-pim**	0.027 (0.31)
**CC1/2**	0.99 (0.76)
**Refinement**	
Reflections used in refinement	40577 (2746)
Reflections used for R-free	2000 (136)
R-work	0.1826 (0.2430)
R-free	0.2145 (0.2877)
Number of non-hydrogen atoms	2308
macromolecules	2016
ligands	0
solvent	292
Protein residues	258
RMS(bonds)	0.010
RMS(angles)	1.04
Ramachandran favored (%)	100.00
Ramachandran allowed (%)	0.00
Ramachandran outliers (%)	0.00
Rotamer outliers (%)	0.45
Clashscore	1.74
Average B-factor	27.34
macromolecules	26.40
solvent	33.83

Statistics for the highest-resolution shell are shown in parentheses.

Supplemental Table S2. Strains used in this study

Strains	Description and genotype	Source/References
Escherichia coli DH5α	F-, Δ(*argF*-lac)U169 *phoA supE44 lacZ*ΔM15 *recA relA endA thi hsdR gyr*	Laboratory collection
Escherichia coli BL21(DE3)	*dcm ompT hsdS gal* λ *DE3*	Laboratory collection
Escherichia coli W3110	F-, lambda- IN(*rrnD-rrnE*)1 *rph*-1	Laboratory collection
Saccharomyces cerevisiae W303	*MATa/MATα {leu2-3,112 trp1-1 can1-100 ura3-1 ade2-1 his3-11,15} [phi$^+$]*	Laboratory collection

Supplemental Table S3. Plasmids used in this study

Plasmid	Description	Source/References
pNDM220	Mini-R1, single-copy vector, LaqI^q, $P_{A1/04/03}$, Amp^R	Gotfredsen and Gerdes, 1998
pBAD33	Expression vector, AraC, arabinose-inducible, Cm^R	Guzman et al., 1995
pNDM220-ARC	*P. ananatis* LMG 20103 *PANA_2924* C-terminal domain cloned into pNDM220	This study
pBAD-ARCimm	*P. ananatis* LMG 20103 *PANA_2923* cloned into pBAD33	This study
pNDM220-ARC-W273A	Trp273-to-Ala substitution in pNDM220-ARC	This study
pNDM220-ARC-E299A	Glu299-to-Ala substitution in pNDM220-ARC	This study
pNDM220-ARC-W323A	Trp323-to-Ala substitution in pNDM220-ARC	This study
pNDM220-ARC-E356A	Glu356-to-Ala substitution in pNDM220-ARC	This study
pRS416_Gal1	URA3/Amp , EN6/ARSH4, GAL1 promoter	Mumberg et al., 1994
pRS416_Gal1-ARC*	*PANA_2924* C-terminal domain cloned into pRS416_Gal1 and containing a 1 bp (A) deletion at position 435	This study
pET-Duet1	pBR322 ColE1 *ori,* T7 promoter, Amp^R, 6×his tag and TEV cleavage site	Novagen
pET-hisTEV-ARC-ARCimm	*P. ananatis* LMG 20103 *PANA_2924* C-terminal domain and *PANA_2923* cloned into pET-Duet1	This study
pET-hisTEV-ARC-E356A	*P. ananatis* LMG 20103 *PANA_2924* C-terminal domain carrying Glu356-to-Ala substitution cloned into pET-Duet1	This study
pRSF-Duet1	RSF1030 (NTP1) *ori*, T7 promoter, Kan^R	Novagen

pRSF-ARCimm	*P. ananatis* LMG 20103 *PANA_2923* cloned into pRSF	This study
pCDF-Duet1	CloDF13 *ori*, T7 promoter, $Strep^R$	Novagen
pCDF-ARCimm	*P. ananatis* LMG 20103 *PANA_2923* cloned into pCDF	This study
pNDM220-LisARC	*L. monocytogenes LS1292* LXG C-terminal domain cloned into pNDM220	This study
pBAD-LisARCimm	*L. monocytogenes LS1292* immunity against ARC cloned into pBAD33	This study
pCH10163	Plasmid containing *E. coli* EC93 *cdiB* and *cdiA* under their natural promoter	Willett et al., 2015
pCH-Cdi::*treX-triX*	*Xenorhabdus boviennii* SS-2004 *treX-triX* cloned into pCH10163	Dumont et al., 2024
pCH10163-ARC-ARC-imm	*P. ananatis* LMG 20103 *PANA_2924* C-terminal domain and *PANA_2923* cloned into pCH10163	This study

Supplemental Table S4. Primers used in this study

Primer name	Sequence 5'- 3'	Purpose
R-pNDM220-seq	TCTTCGCTATTACGCCAGCT	Check cloned sequences in pNDM220 vector
pBAD Fwd	TCCATAAGATTAGCGGATCC	Check cloned sequences in pBAD33 vector
5-pNDM-PANAtox-KpnI	GCTA**GGTACC**ATGGCAGCCTTTAATGCGTGGCG	Clone *P. ananatis* LMG 20103 *PANA_2924* C-terminal domain into pNDM220 through *KpnI* and *EcoRI* sites
3-pNDM-PANAtox-EcoRI	GCTA**GAATTC**TTAGTTATCCCTTACAAAAATGACTTTTTCCGTCTGTG	
5-pBAD-PANAimm-SalI	GCTA**GTCGAC**ATGAAAATTACAGGCACAAGTTCGTATGTGATG	Clone *P. ananatis* LMG 20103 *PANA_2923* into pNDM220 through *SalI* and *HindIII* sites
3-pBAD-PANAimm-HindIII	GCTA**AAGCTT**TTATACAAATTTCAGGACCATATGAGATCCTTGGG	
F-paARC-Xba	CCCCC**TCTAGA**ACTAGTGGATCCATGGCAGCCTTTAATGCGTGGCGTAA	Clone *P. ananatis* LMG 20103 *PANA_2924* C-terminal domain into pRS416_Gal1 through *KpnI* and *XbaI* sites
F-pRS416	CAACATTTTCGGTTTGTATTACTTC	Check cloned sequences in pRS416_Gal1 vector
A-ARC-Pa-W33A	GCGTCAGGGCGCACTGAAGG	W273A substitution in *P. ananatis* LMG 20103 *PANA_2924*
B-ARC-Pa-W33A	GAAAAAGGCACTGTCCGGATCAG	
A-ARC-Pa-E59A	GCATCCACAATCAAAGATAAAAATATTAAAATGCCTG	E299A substitution in *P. ananatis* LMG 20103 *PANA_2924*
B-ARC-Pa-E59A	GAGCGTGACACCGCCT	

A-ARC-Pa-W83A-2	CAGAGGATGTCTCCGCGTCT TATG	W323A substitution in *P. ananatis* LMG 20103 *PANA_2924*
B-ARC-Pa-W83A-2	CAGCCTTGATGCTCTGCGGG TTATC	
A-ARC-Pa-E116A	GCGTTACCGCGCCTGATGGG CAATG	E356A substitution in *P. ananatis* LMG 20103 *PANA_2924*
B-ARC-Pa-E116A-2	AACGTTTTCCCATATATTAC CTTCACGCAG	
F-PaARC-Bmt	GGGC**GCTAGC**GCAGCCTTT AATGCGTGGCGTAA	Clone *P. ananatis* LMG 20103 *PANA_2924* C-terminal domain and *PANA_2923* into pET-Duet1-his-TEV through *BmtI* and *HindIII* sites
R-PaImm-Hind	GACT**AAGCTT**TTATACAAAT TTCAGGACCATATGAGATCC	
F-PaImm-Sac	GGAG**GAGCTC**ATGAAAATT ACAGGCACAAGTTCG	Clone *P. ananatis* LMG 20103 *PANA_2923* into pRSF-Duet1 and pCDF-Duet1 through *SacI* and *HindIII* sites
ACYCDuetUP1	GGATCTCGACGCTCTCCCT	Check cloned sequences in pET-Duet1-his-TEV, pRSF-Duet1, and pCDF-Duet1 vectors
Duet_down1	GATTATGCGGCCGTGTACAA	
5-pNDM-Lis-ARCtox-kpn	GCTA**GGTACC**ATGGCTAAA GGATTTAATATTGACGAAAT TCATCC	*Clone L. monocytogenes* LS1292 LXG C-terminal domain into pNDM220
3-pNDM-Lis-ARCimm-EcoRI	GCTA**GAATTC**TTACTTCCTT TCAAAAATTATTTTTCAAG TCC	
F-Lis-ARCimm-sac	GGAG**GAGCTC**ATGAAAATT ACAGGTAATAGTTCACATGT G	Clone *L. monocytogenes* LS1292 immunity against ARC into pBAD33
R-Lis-ARCimm-xba	GACT**TCTAGA**TTATTCAAAT GAAATTTGTACTGTATTTTC ATTTGTG	
F_CDI_plasmid	CCCAAAGGTTAGACACCAG ACC	Amplify CDI part from pCH-Cdi::*treX-triX*
R_CDI_plasmid	TGTCGCTGTATTGCGACTCA T	
F_ARCoperon_CDI_mega	ATGAGTCGCAATACAGCGA CAGCAGCCTTTAATGCGTGG CGTAAC	Amplify the *PANA_2924* C-terminal domain and *PANA_2923* for fusion with CDI components
R_ARCoperon_CDI_mega	GGTCTGGTGTCTAACCTTTG GGTTATACAAATTTCAGGAC CATATGAGATCCTTGGG	
pCH3_F	CTTCTGATCCTGAAAATCAG GCCATG	Check cloned sequences in pCH10163-ARC-ARC-imm construct
pCH3_R	CCTTGCTACAGATTTCAGAC TCAACTCTC	
